# A compact motorized end-effector for ankle rehabilitation training

**DOI:** 10.3389/frobt.2024.1453097

**Published:** 2024-08-28

**Authors:** Renxiang Wu, Mingyang Luo, Jiaming Fan, Jingting Ma, Naiwen Zhang, Jianjun Li, Qiuyuan Li, Fei Gao, Guo Dan

**Affiliations:** ^1^ Medical Electronic Instrument Transformation Engineering Technology Research Center of Guangdong Province, Shenzhen, China; ^2^ School of Biomedical Engineering, Health Science Center, Shenzhen University, Shenzhen, Guangdong, China; ^3^ Rehabilitation Clinic, Shenzhen University General Hospital, Shenzhen, Guangdong, China; ^4^ Guangdong Provincial Key Lab of Robotics and Intelligent System, Shenzhen Institute of Advanced Technology, Chinese Academy of Sciences, Shenzhen, Guangdong, China

**Keywords:** ankle rehabilitation robot, ROM rehabilitation, stroke, motion intent recognition, lower limb rehabilitation robot

## Abstract

This paper introduces a compact end-effector ankle rehabilitation robot (CEARR) system for addressing ankle range of motion (ROM) rehabilitation. The CEARR features a bilaterally symmetrical rehabilitation structure, with each side possessing three degrees of freedom (DOF) driven by three independently designed actuators. The working intervals of each actuator are separated by a series connection, ensuring they operate without interference to accommodate the dorsiflexion/plantarflexion (DO/PL), inversion/eversion (IN/EV), and adduction/abduction (AD/AB) DOF requirements for comprehensive ankle rehabilitation. In addition, we integrated an actuator and foldable brackets to accommodate patients in varied postures. We decoded the motor intention based on the surface electromyography (sEMG) and torque signals generated by the subjects’ ankle joints in voluntary rehabilitation. Besides, we designed a real-time voluntary-triggered control (VTC) strategy to enhance the rehabilitation effect, in which the root mean square (RMS) of sEMG was utilized to trigger and adjust the CEARR rehabilitation velocity support. We verified the consistency of voluntary movement with CEARR rehabilitation support output for four healthy subjects on a nonlinear sEMG signal with an $R^{2}$ metric of approximately 0.67. We tested the consistency of triggering velocity trends with a linear torque signal for one healthy individual with an $R^{2}$ metric of approximately 0.99.

## 1 Introduction

The ankle joint complex (AJC) is crucial for individuals acquiring locomotion function. The interaction between the ground and the human body is directly decided by the ankle movement, which significantly affects the stability of walking. Lower limbs’ movements are controlled by the central nervous system (CNS) (Nishikawa et al., 2007). Thus, neurological disorders such as stroke can affect the human CNS that may lead to lower extremity dysfunction (Díaz et al., 2011; Chen et al., 2016). It is reported that approximately 50% of stroke patients’ ankle suffer from a restricted motion, in which the range of motion (ROM) of patients’ ankles is significantly decreased, relative to that of the healthy (Tyson et al., 2013; Gorst et al., 2019; Kim et al., 2023). To avoid ankle and foot deformities or ankle joint stiffness, motor rehabilitation with assistive devices is performed in the early phase of stroke (Hsu et al.2003; Chung et al., 2004; Lin et al., 2006; Roy et al., 2013). The ankle motor rehabilitation process can be divided into three stages: early functional rehabilitation (aiming to help stroke patients gradually regain normal ROM and increase muscle strength), intermediate functional rehabilitation (improving balance and proprioception), and advanced functional rehabilitation (restoring advanced functions such as walking and jumping) (Yoon et al., 2006). Restoring joint ROM and coordination in the ankle joint is one of the critical factors in regaining independent and safe mobility (Zhai et al., 2021; Li J. et al., 2023).

Ankle ROM rehabilitation by physical therapists (PTs) is a widely accepted approach in conventional practices (Komada et al., 2009). To reduce human effort and rehabilitation time, ankle rehabilitation robots are designed to assist patients in engaging in repetitive and high precision training that cannot be achieved by PTs’ intervention easily (Yu et al., 2013). In addition, ankle rehabilitation robots can automatically adjust training strategies based on patients’ conditions to improve rehabilitation performance. In (Cooke and Bliss, 2006), it is reported that the rehabilitation process can improve the function and structure of injured nerves in stroke patients, thus improving ankle motor function and allowing patients to recover ankle motor function.

The existing ankle rehabilitation robots can be categorized as exoskeletons for mobile rehabilitation and end-effector robots for fixed rehabilitation (Dong et al., 2021b). The AJC possesses three degrees of freedom (DOF), including dorsiflexion/plantarflexion (DO/PL), adduction/abduction (AD/AB), and inversion/eversion (IN/EV) (Dong et al., 2021a). To fully restore the function of a healthy ankle, the ankle rehabilitation robot should cover the 3-DOF at least. Ren. Y et al. developed an exoskeleton robot for ankle rehabilitation training while lying in bed, which only supported rehabilitation training in one DOF of ankle dorsiflexion/plantarflexion (DO/PL) (Ren et al., 2017). The ankle-foot orthoses’ approximate construction similarly ignores support for ankle DO/PL out of degrees of motion rehabilitation (Ferris et al., 2006; Rodriguez Hernandez et al., 2023). Wang. T et al. introduced an ankle rehabilitation exoskeleton tailored for post-stroke patients (Wang et al., 2021), which can automatically align with the user’s ankle rotation center. It offers a 3-DOF rehabilitation scope, and the AD/AB rotation is a coupled motion.

The structure of the end-effector rehabilitation robot may yield superior results in certain scenarios compared to an exoskeleton during rehabilitation training (Bruni et al., 2018; Molteni et al., 2018). It is better suited for diverse patients (Girone et al., 2001; Liu Q. et al., 2018; Wang et al., 2022), as it engages only one segment of the affected limb, mobilizing the target joint without impeding the movement freedom of the limb’s other joints. Girone et al. developed the Rutgers Ankle system (Girone et al., 2001), modeled on the Stewart platform architecture, utilizes six commercial pneumatic cylinders as its actuators. These cylinders facilitate 6-DOF rehabilitation of the ankle joint, but the center of rotation may not be consistent with the center of the ankle joint, which might cause secondary injuries (Wang et al., 2022). Prashant et al. proposed the adaptive wearable parallel 3-DOF robot with pneumatic muscle actuators (PMA) for treating ankle injuries (Jamwal et al., 2014). PMAs are compliant but nonlinear actuators. Because of that, it is quite hard for realizing tracking control precisely in practice. Wang. C et al. presented a 3-RUS/RRR redundantly actuated parallel ankle rehabilitation robot (Wang et al., 2013).

Bilateral limb coordination is crucial in the rehabilitation journey of stroke patients (Whitall et al., 2000; Cauraugh and Summers, 2005). Bilateral ankle coordination is essential for walking and maintaining balance in everyday activities. Chang. J et al. indicated that bilateral synergistic rehabilitation is more efficacious than unilateral approaches (Chang et al., 2023). Moreover, most work illustrated that rehabilitation training with patients’ voluntary participation can promote neurological reconstruction and motor function recovery more effectively than passive rehabilitation (Rizzolatti and Craighero, 2004; Cooke and Bliss, 2006; Thieme et al., 2013). For stroke patients with muscle dysfunction and cognitive problems, rehabilitation robots should firstly recognize motion intent and then properly program rehabilitation training tasks and human-machine interaction (Pareek et al., 2019). Different signals including plantar pressure (Zhu et al., 2022), motion angle (Gao et al., 2020; Zhu et al., 2022), surface electromyography (sEMG) (Li K. et al., 2020), electroencephalogram (EEG) (Liu D. et al., 2018), and torque signals (Feng et al., 2019), are analyzed to decode the patient’s motion intent. sEMG can directly reflect the motion of the corresponding muscles (Zhang et al., 2021), which can be detected before 30–150 ms when the associated limb movement occurs (Li X. et al., 2023). sEMG-based motion intent control can be divided into continuous control and triggered control (Meng et al., 2015). In early ROM rehabilitation in AJC, the task did not require a complex motor pattern, namely, it did not require motor trajectory planning and complex control. Continuous control systems generally require complex controllers, employing machine learning, to handle the nonlinearity of sEMG signals, along with individual and temporal variabilities, necessitating training on diverse datasets. However, the real-time performance of certain regression models is suboptimal (Xiao et al., 2023), diminishing the advantage of sEMG signals’ velocity relative to limb movement. Triggered control can avoid the above problems through simple numerical judgment (Tian et al., 2022), This approach is appropriate for the aforementioned signals and concurrently adheres to the design principle of simplicity in control systems for rehabilitation robots.

This paper developed a compact end-effector ankle rehabilitation robot (CEARR). Some preliminary work has been disclosed in our previous conference paper (Wu et al., 2023). The feature of the CEARR include facilitating simultaneous bilateral ankle rehabilitation and encompassing the 3-DOF essential for ankle recovery. The system is designed with three distinct actuators, each equipped with motor drives, to support the rehabilitation of a specific DOF of ankle joint. These actuators engage through a gear and rack mechanism and are combined in series from bottom to top. Additionally, the rotation center for this 3-DOF rehabilitation can align with the ankle joint, allowing pose adjustment for use in either a supine or seated position for stroke patients. We designed a voluntary-based trigger control (VTC) strategy, which enables the system to acquire torque and sEMG signals from the user during rehabilitation to analyze the user’s motion intent and provide appropriate rehabilitation. The system employs a rehabilitation program developed using Unity, offering visual feedback and directing patients toward correct rehabilitation movements throughout their recovery process. Finally, we assess the performance of the ankle rehabilitation robot by conducting tests on healthy subjects while they sit and train with the system.

## 2 Methodology

### 2.1 System overview

**FIGURE 1:**
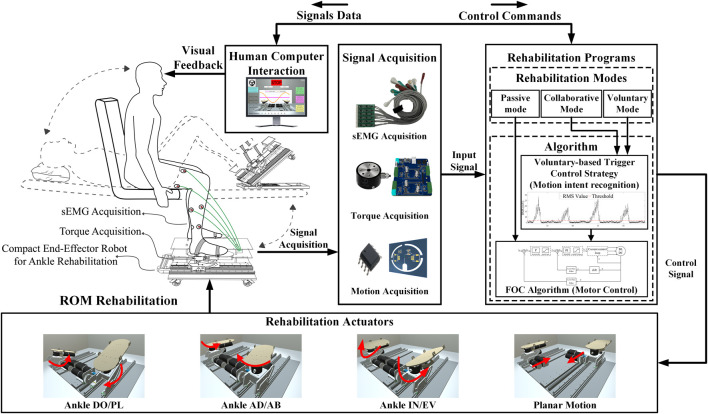
CEARR system is designed for ankle rehabilitation training. It consists of four main parts: CEARR rehabilitation actuators, signal acquisition, rehabilitation programs, and visual biofeedback human-computer interaction.

The configuration of the CEARR system presented in this study is depicted in Figure 1. It consists of four main parts: CEARR rehabilitation actuators, signal acquisition, rehabilitation programs, and visual biofeedback human-computer interaction. The CEARR Based on the VTC strategy identifies the user’s motion intent by analyzing features of the user’s torque or sEMG signals. Then, the system employs a field-oriented control (FOC) algorithm to control four actuators for adjusting the device’s initial posture and the human ankle’s 3-DOF motion according to preset rehabilitation programs. The motor motion data and rehabilitation condition are also collected and transmitted to a human-computer interaction platform, offering visual feedback.

### 2.2 Mechanism design

**FIGURE 2:**
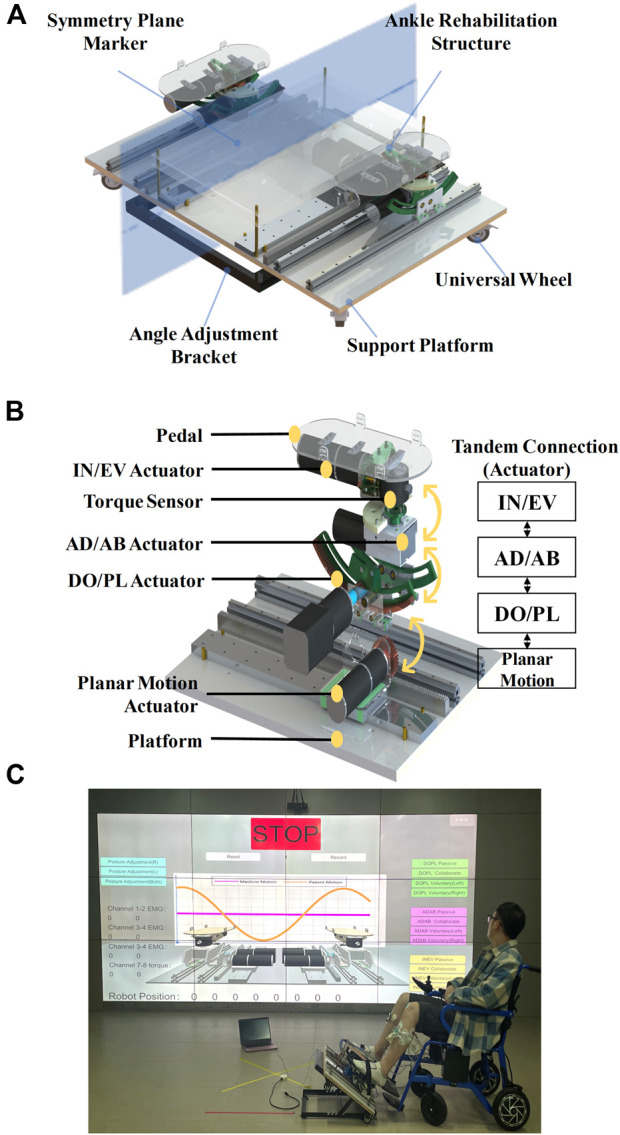
Mechanical design of the CEARR. **(A)** 3D model of the CEARR prototype, **(B)** Drive units for each movement. **(C)** The subject is equipped with the CEARR for rehabilitation training.

We designed the CEARR for patients who can place their feet on the robot’s end pedals and secure them using straps for motor rehabilitation. The design of the platform-based structure guarantees proper support for the end-effector and facilitates adjustments in the robot’s working posture. These adjustments are achieved by foldable adjustment brackets modifying the platform tilt angle, which caters to the diverse postures of stroke patients with varying rehabilitation requirements. The foldable adjustment bracket and four universal wheels were incorporated into the platform base to easily adjust the CEARR’s angle and position. The mechanical design of the CEARR system is depicted in Figure 2. The CEARR system facilitates rehabilitation by enabling ankle joint movements, including DO/PL, IN/EV, and AD/AB. CEARR features a single planar motion DOF, assisting patients in modifying their leg posture throughout the ankle movement process. The motion of each DOF is shown in Figure 1. The robot’s motion actuators are designed in tandem and avoid singularity in the workspace. This tandem structure also facilitates the adjustment of the structure and does not affect each other. The bilaterally symmetrical mechanical structure ensures effective support for rehabilitation training on the robot, supporting to patients with hemiplegia on any side. It also enables bilateral collaborative rehabilitation to ensure balanced muscle function on both sides and provides unilateral rehabilitation support for patients experiencing hemiplegia on varying sides. The robot prototype is driven by a Brushless Direct Current (BLDC) motor (Size: 600mm×500mm×215mm ; Weight: 20kg ). And is shown in Figure 2B.

The motion mechanism is composed of four distinct actuators: a planar motion actuator, an ankle DO/PL actuator, an ankle AD/AB actuator, and an ankle IN/EV actuator. The four actuators are connected in tandem from bottom to top according to the sequence given above: The planar actuator is linked to the ankle DO/PL actuator through an L-shaped connector secured to the slider on the rail. The ankle DO/PL actuator and the ankle AD/AB actuator are directly interconnected via the BLDC motor. The ankle AD/AB actuator and the ankle IN/EV actuator are concurrently integrated, utilizing distinct fixation units in conjunction with the end-effector pedal, ultimately achieving the tandem connection of each actuator to minimize the robot’s dimensions and maintain compactness, an internal gear design is employed. No supplementary linkage structure is incorporated for each mechanism.

The planar actuator employs a rack and pinion meshing drive in conjunction with a slider to facilitate movement relative to above the platform. The ankle DO/PL actuator employs a planar gear transmission. To minimize friction within the motion mechanism during movement, bearings are added tangentially to the rotating and supporting structures, transforming sliding friction into rolling friction. A similar approach is applied to the ankle IN/EV transmission mechanism. Meanwhile, the ankle AD/AB actuator utilizes a worm gear drive.

On each side of the CEARR, every actuator is powered by an individual BLDC motor drive. Regarding the drive motors utilized in the actuators, two 25W BLDC motors (MS32RBL-32, Shantou Mseag Technology Co., Ltd, China) are employed for the planar motion actuators, combined with a 1:51 planetary reduction gear. Two 40W BLDC motors were chosen for the ankle DO/PL motion structure, combined with a 1:115.9 planetary reduction gear. The AD/AB and IN/EV actuators use the same motors as the planar motion actuators, with reduction ratios 1:290 and 1:51, respectively.

**FIGURE 3:**
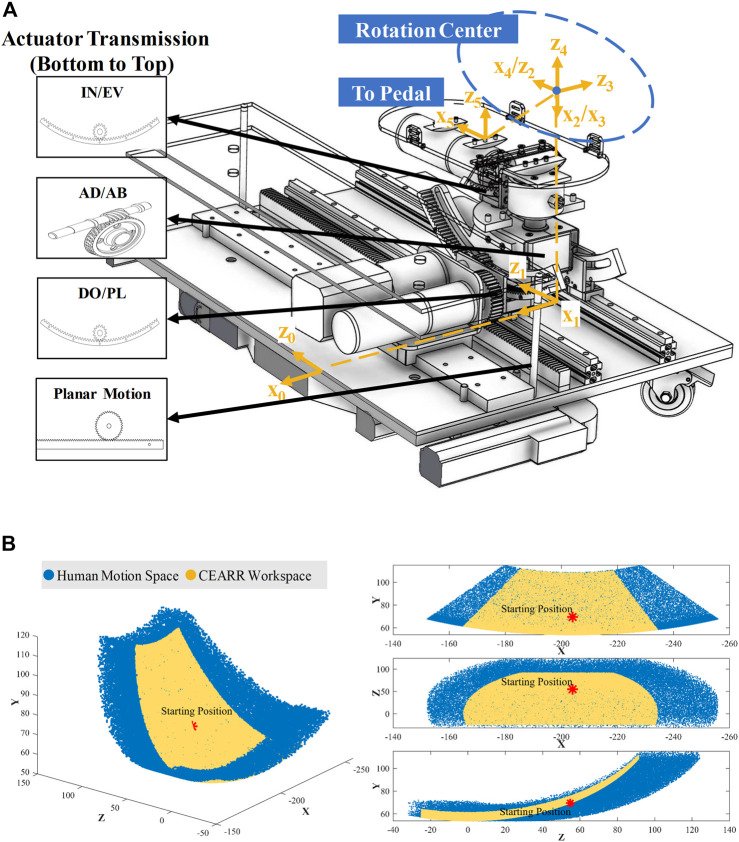
Workspace analysis of CEARR. **(A)** SDH model and the coordinates of each DOF. **(B)** Comparison of maximum workspace for CEARR and healthy people.

To ensure the safety of the patient’s ankle joint during rehabilitation training, it is imperative to ensure that the center of rotation of the pedals aligns with the center of rotation of the ankle joint (Liu Q. et al., 2018; Li et al., 2020a; Wang et al., 2022). The rotational axes of the CEARR for the DO/PL and IN/EV actuators are defined by internal gears corresponding to the radius, with the AD/AB actuator’s rotation being perpendicular to the pedal. The intersection of the rotational axes of the three CEARR actuators is positioned above the pedal and is vertically adjustable to align with the center of rotation of the ankle joint. The rotational axes of the three actuators are the same as the center of rotation of the ankle joints, as shown in Figure 3A. The modified CEARR operating angle range of each actuator is compared with the healthy human AJC ROM as shown in Table 1.

**TABLE 1:** Motion range of CEARR and healthy people.

Motion type	CEARR design	Healthy human (Li et al., 2020)
Ankle DO/PL	0−30°/0−50°	20.3°−29.8°/37.6°−45.8°
Ankle IN/EV	0−30°/0−30°	14.5°−22.0°/10.0°−17.0°
Ankle AD/AB	0−40°/0−30°	22.0°−36.0°/15.4°−25.9°

where $a_{1}=-204\;mm$ , $a_{2}=-157.6\;mm$ , $a_{5}=54.80\;mm$ , $d_{5}=-88.20\;mm$ .

### 2.3 Kinematic analysis

The ROMs for each DOF in human ankle are listed in Table 1. To fully reproduce the motion of the human ankle, CEARR’s ROM should cover the human ankle’s ROM. We employed a modified Standard Denavit-Hartenberg (SDH) model to model the CEARR, in which the 3-DOF are defined as ball joints thus enabling the joint’s center to align with the rotation center of the AJC. Based on the SDH principle for the homogeneous transformation, the $T_{i}$ transformation process can be pressed by Eq. 1:

$$\begin{array}{l} T_{i}={\mathrm{Rot}}_{z,\theta_{i}}{\mathrm{Trans}}_{z,d_{i}}{\mathrm{Trans}}_{x,a_{i}}{\mathrm{Rot}}_{x,\alpha_{i}}\\ \;=\left[\begin{array}{cccc} {\cos\theta }_{i}\; & {‐\sin\theta }_{i}{\cos\alpha }_{i}\; & {‐\sin\theta }_{i}{\sin\alpha }_{i}\; & \alpha_{i}{\cos\theta }_{i}\\ {\sin\theta }_{i}\; & {\cos\theta }_{i}{\cos\alpha }_{i}\; & ‐{\cos\theta }_{i}{\sin\alpha }_{i}\; & \alpha_{i}{\sin\theta }_{i}\\ 0\; & {\sin\alpha }_{i}\; & {\cos\alpha }_{i}\; & d_{i}\\ 0\; & 0\; & 0\; & 1 \end{array}\right] \end{array}$$ (1)

In SDH model case, the transformation matrix from frame link i to link i+1 can be expressed by four parameters. The parameters $a_{i}$ , $\alpha_{i}$ , $d_{i}$ and $\theta_{i}$ are referred to as link length, link twist, link offset, and joint angle, respectively. The parameters derived from the CEARR’s SDH model are shown in Table 2. $T_{5}^{0}$ represents the forward kinematic description for one side of the CEARR as shown in Eq. 2:

$$\begin{array}{l} T_{5}^{0}=T_{1}^{0}∙T_{2}^{1}∙T_{3}^{2}∙T_{4}^{3}∙T_{5}^{4}\\ \;=\prod_{i=1}^{5}\left[\begin{array}{llll} {\cos\theta }_{i} & {-\sin\theta }_{i}{\cos\alpha }_{i} & {-\sin\theta }_{i}{\sin\alpha }_{i} & \alpha_{i}{\cos\theta }_{i}\\ {\sin\theta }_{i} & {\cos\theta }_{i}{\cos\alpha }_{i} & -{\cos\theta }_{i}{\sin\alpha }_{i} & \alpha_{i}{\sin\theta }_{i}\\ 0 & {\sin\alpha }_{i} & {\cos\alpha }_{i} & d_{i}\\ 0 & 0 & 0 & 1 \end{array}\right] \end{array}$$ (2)

**TABLE 2:** SDH parameters of CEARR.

Link	$\alpha_{i}$	$a_{i}$	$d_{i}$	$\theta_{i}$	Range (^*^)
1	0	$a_{1}$	0	0	0
2	0	$a_{2}$	$d_{2}^{*}$	−90°	0‐33cm
3	90°	0	0	$\theta_{3}^{*}$	−30‐30°
4	−90°	0	0	$\theta_{4}^{*}+90{}^{\circ}$	−50‐30°
5	0	$a_{5}$	$d_{5}$	$\theta_{5}^{*}$	−30‐40°

We utilized the Robotics Toolbox for MATLAB, in tandem with the Monte Carlo method, to compute the workspaces of CEARR as well as the healthy human ankle. The CEARR to encompass the ROM rehabilitation workspace required for stroke patients as shown in Figure 3B.

### 2.4 Signal acquisition

The system uses CY8C5888LTILP097 and STM32F103RCT6 as the main controller, and the low-level motor drivers are ESP-32. The system acquires sEMG signals, torque signals, and motor rotor rotation angle signals, which will be used for detecting lower limb motion intent and also as feedback for the control of BLDC motors. A tailor-made six-channel sEMG differential acquisition module was employed to collect sEMG signals from the three muscles responsible for the four distinct movements of the ankle joint, thereby negating the need for motion classification during identification. Gastrocnemius and Halibut muscles are in charge of the plantarflexion. Tibialis anterior drives the dorsiflexion and inversion. Peroneus longus actuates the eversion. Two torque sensors are used to acquire the torque signals generated during ankle joint AD/AB rehabilitation. Absolute magnetic encoders (AS5600, AMS, Austria) were used to measure the rotation motion of each BLDC rotor.

All signals are sampled at a rate of 1 kHz, and the data is transmitted using a straightforward communication protocol. The system hardware signal acquisition torque and sEMG acquisition module are directly connected to the main controller board, and the motor angle signal acquisition module is connected to the driver board of each motor. BLDC motor driver is designed according to SimpleFOC open-source project (Skuric et al., 2022). Each motor driver board can drive the motors of the bilateral actuators at the same time to meet the requirements of the bilateral collaborative mode described below. The four driver boards send the motor angle signals to the main controller board, and the main control board integrates the torque, sEMG, and motion signals and sends them to the upper computer.

### 2.5 Control strategy

**FIGURE 4:**
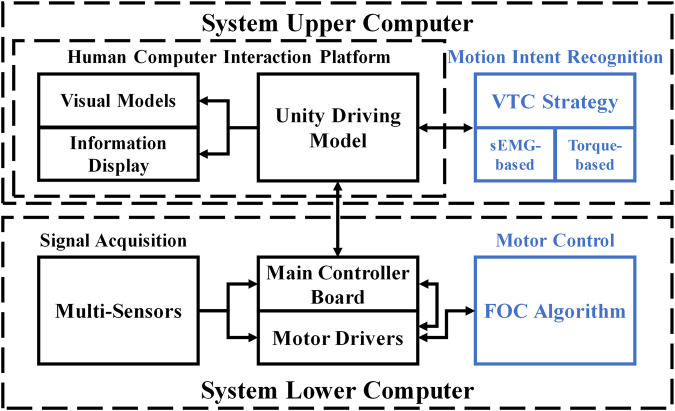
System configuration of the CEARR.

The control framework for the system is composed of the FOC algorithm for controlling BLDC motors, the VTC strategy for motion intent recognition, and the strategy based on sEMG signals or torque signal. The configuration can be depicted in Figure 4. FOC algorithms are commonly employed for efficient control of BLDC motors (Mohanraj et al., 2022). FOC enables precise control over the magnitude and direction of the motor’s magnetic field and, at the same time, can have a motor with high-velocity dynamic response, smooth torque, and low noise during the movement. The FOC consists of three control loops that operate from the outside to the inside: the position loop, the velocity loop, and the current loop. In particular, the current loop process decomposes the stator current of the motor and independently controls the decomposed currents in order to accurately output the necessary motor operating current. This enables precise control of the torque, velocity, and position of the BLDC motor. The rehabilitation mode of the system will be designed based on FOC.

**FIGURE 5:**
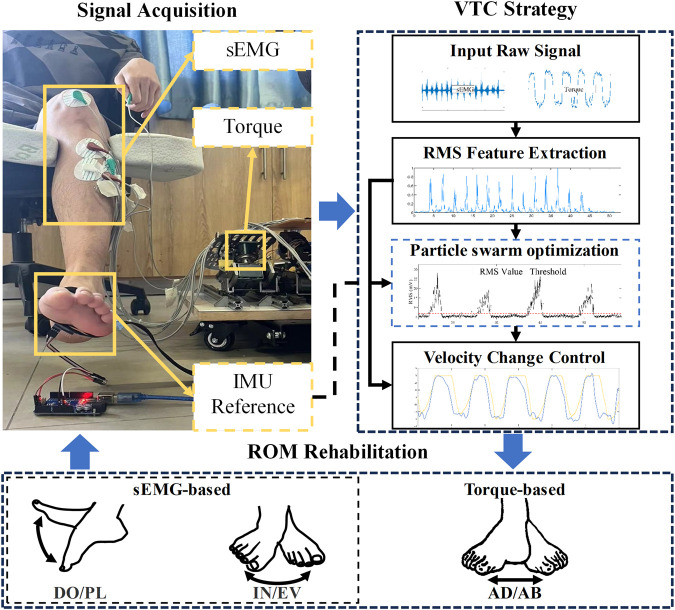
The VTC strategy workflow and subsequent experimental reference angle acquisition schematic.

The implementation of the VTC strategy for CEARR is depicted in Figure 5. The VTC strategy operates in both bilateral collaborative mode and voluntary mode. The VTC strategy extracts the root mean square (RMS) features from the sEMG or torque signals of patients during rehabilitation training, characterizing their voluntary participation due to its real-time effectiveness (Ma et al., 2019). Upon reaching the trigger threshold, the input RMS value indicates the development of voluntary motion intent in the patient, prompting CEARR to provide support for variable-velocity rehabilitation training based on RMS quantification. Conversely, when the value falls below the threshold, the robot ceases rehabilitation training. F For motions involving different 3-DOF, these are decomposed into a total of six directional velocity changes. Each directional VTC strategy is characterized by the same velocity equation, as shown in Eq. 3:

$$\left\{\begin{array}{l} V_{i}={Vvtc}_{i}\;{\;RMS}_{i}\geq T_{t}\\ V_{i}=0\;{\;RMS}_{i}\leq T_{t} \end{array}\right.$$ (3)

where $V_{i}$ is the instantaneous angular velocity of the sampled value at the i moment, $T_{t}$ is the mean of the RMS value of the torque or sEMG of the patient’s simple movements for the first 30 s using CEARR. ${RMS}_{i}$ is the windowed RMS value of the signal at moment i . ${Vvtc}_{i}$ is the velocity change curve based on the sigmoid function designed from the RMS features of signals, as shown in Eq. 4:

$${Vvtc}_{i}=\frac{k}{1+e^{-q\left(\left|{RMS}_{i}-T_{t}\right|\right)+b}}$$ (4)

Where k , q are the velocity proportional gain and input signal gain respectively, they provide the gain that approximates the nonlinear velocity change of human limb movement. b is the parameter that adjusts the minimum velocity post-triggering. The higher b is, the greater the initial velocity upon triggering. With b set to 0, the minimum velocity for ${Vvtc}_{i}$ triggering becomes k/2 . Additionally, the sigmoid function component effectively limits the velocity to a range between 0 and k . This approach safeguards patient rehabilitation by protecting against excessive velocity changes, especially in cases of high ${RMS}_{i}$ values. This design functions as a feedback incentive for voluntary participation, providing higher velocities for high ${RMS}_{i}$ values and lower velocities for low ${RMS}_{i}$ values. To ensure CEARR’s velocity gain aligns with the motion trend of the ankle joint, the first 30 s of torque and sEMG data (need inertial measurement unit (IMU) as reference) acquired during the initial use of CEARR by patients are used. These data are then optimized using particle swarm optimization (PSO) to refine the above gains. The optimization of the fitness function involved setting an appropriate function to balance the coefficient of determination ( $R^{2}$ ) and root mean square error (RMSE), as shown in Eqs 5, 6:

$$R^{2}=1-\frac{\sum_{n=0}^{N}{\left(y_{i}-{\hat{y}}_{i}\right)}^{2}}{\sum_{n=0}^{N}{\left(y_{i}-\hat{y}\right)}^{2}}$$ (5)

$$\text{RMSE}=\sqrt{\frac{1}{N}\sum_{n=1}^{N}{\left(y_{i}-{\hat{y}}_{i}\right)}^{2}}$$ (6)

where $y_{i}$ is the estimated value, ${\hat{y}}_{i}$ is the measured value, N is the number of samples, $\hat{y}$ is the mean of the measured value. The closer the $R^{2}$ is to one and the closer RMSE is to 0, the better the performance of the model. The fitness function is set as shown in Eq. 7:

$$fitness=\text{RMSE}-c\times R^{2}$$ (7)

where c is the scale factor, which is the share of $R^{2}$ in the fitness function, and was determined to be c=10 after several trials. The above PSO approach as illustrated in Algorithm 1. The final angular output of each actuator in CEARR is determined by integrating the VTC strategy through the subtraction of the corresponding two action velocities. The specific positive and negative directions are calibrated according to the SDH model, as shown in Eq. 8:

$$\theta_{n}=\sum_{i=1}^{n}\left({Vp}_{i}+{Vn}_{i}\right)$$ (8)

where ${Vp}_{i}$ is motion in the positive direction of an individual actuator, such as motion in the DO direction performed on a DO/PL actuator, ${Vn}_{i}$ is motion in the negative direction, such as PL.

### 2.6 Rehabilitation modes

The CEARR’s bilaterally symmetrical structure allows for three rehabilitation modes and visual feedback for rehabilitation training. For different stages of stroke rehabilitation, we have designed three different rehabilitation modes. Specifically, for patients with lower muscle strength in the Brunnstrom stages one and two, we adopt a passive mode; for patients who have recovered certain muscle strength in the Brunnstrom stages three and four, we employ a bilateral collaborative mode; for patients in Brunnstrom stages five or above who possess strong independent motor ability, we utilize a voluntary mode (Brunnstrom, 1970).

Algorithm 1 Signal Processing with PSO.

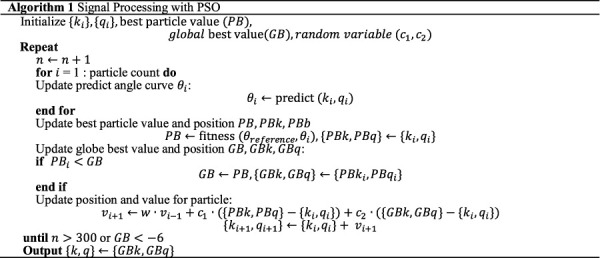

#### 2.6.1 Passive mode

**FIGURE 6:**
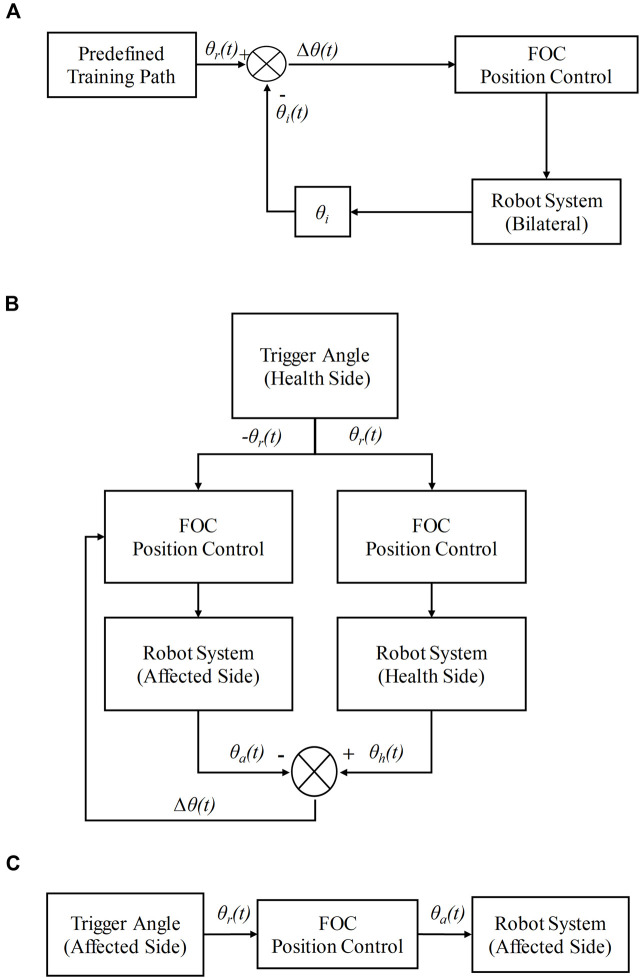
Rehabilitation modes. **(A)** Passive mode, **(B)** Bilateral collaborative mode, **(C)** Voluntary mode.

In passive mode, the CEARR system entirely drives the patient’s ankle joint for ROM rehabilitation. This mode is predominantly designed for early-stage stroke patients with minimal voluntary muscle contractions or restricted voluntary movements. Continuous passive movement of the ankle joint promotes enhanced blood circulation and metabolism throughout the limb, preventing muscle spasms. Concurrently, it mitigates muscle atrophy and other conditions stemming from prolonged patient immobility, assisting in the restoration of joint mobility (Veerbeek et al., 2014). The system passive mode control flowchart is shown in Figure 6A. Where $\theta_{i}\left(t\right)$ is the real-time motion angle of the CEARR system, $\theta_{r}\left(t\right)$ is the predefined exercise training ROM angle, $∆\theta \left(t\right)$ is the angular deviation corresponding to the predefined trajectory. The difference between the angle of CEARR’s individual DOF of motion and the predefined ROM angle is used as input to the FOC position controller to ensure that the robot can move on a fixed motion path.

#### 2.6.2 Bilateral collaborative mode

Bilateral collaborative mode involves the patient’s motion intent control of the robot to perform rehabilitation training motion. This mode focuses on the situation where the patient’s healthy limb can perform some mobility exercise movements, but the muscle strength of the affected limb has not been restored. By recognizing the movement intent of the patient’s healthy side to drive the healthy side actuator to move, the affected side actuator, according to the movement of the healthy side actuator, carries out synergistic (isotropic or mirror) movement and finally drives the patient’s affected side to carry out rehabilitation training. The Bilateral collaborative mode control flowchart is shown in Figure 6B.

The sEMG or torque signals are acquired from the healthy side to control the motion and angle of the healthy side. The position control of the healthy side motion mechanism is achieved by calculating the magnitude of the angle value $\theta_{r}\left(t\right)$ based on the regression of the healthy side sEMG signals. On the other hand, the affected-side actuator is controlled using real-time position information from the healthy side actuator.

#### 2.6.3 Voluntary mode

In the voluntary mode, the patient independently controls the motors on the affected side based on their motion intents, without any assistance or hindrance from the system. This mode caters to patients who have regained muscle strength and have the capability for refined motor movements. Self-directed exercise not only aids in muscle strengthening but also facilitates the recovery of the central nervous system (Lotze et al., 2003). The voluntary mode control flowchart is shown in Figure 6C. It should be noted that the bilateral collaborative mode essentially functions as a voluntary-passive hybrid tracking mode, where the healthy side operates in voluntary mode and the affected side synchronizes with the movement of the healthy side.

### 2.7 Visual feedback interface

**FIGURE 7:**
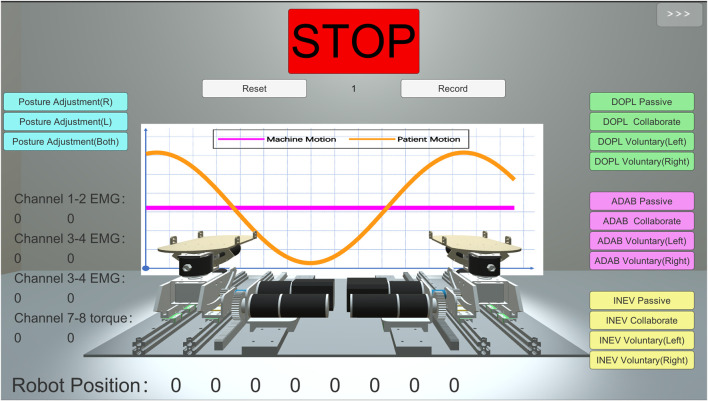
Visual feedback interface of CEARR.

Rehabilitation exercises that actively involve patients often yield superior outcomes in neurological reconstruction and motor function recovery. Designing immersive gaming experiences around this principle has proven to produce noteworthy rehabilitative results (Kiper et al., 2018). F. Noveletto et al. presented that incorporating strategies such as visual feedback can alleviate the monotony of training (Noveletto et al., 2020), bolster patient engagement, and consequently amplify the effectiveness of the training. Therefore, a visual feedback program was developed using Unity. This program can display rehabilitation status and parameters in real-time by communicating with a downstream machine. Additionally, rehabilitation practitioners can flexibly switch patients’ rehabilitation modes via interactive virtual buttons. The system imports CEARR’s CAD model based on Unity, and the whole human-machine interface consists of three parts, namely, the virtual model, the control part, and the information display part. In the heart of the interface lies the virtual machine model, which allows for real-time monitoring of the motion state of the physical-mechanical structure. Additionally, it can provide guidance to patients during their rehabilitation process. To guide the patient to show the force exerted more visually, we added a reference motion curve and an actual motion curve in the background of the machine, respectively. The visual feedback interface is shown in Figure 7.

To maintain consistency in subsequent experiments involving motion intent, an indicator trajectory was incorporated into the interface. Subjects were instructed to attempt to track this motion profile during movement, represented by the Eq. 9:

$$T_{s}=30\sin\left(0.2t\right)$$ (9)

where t is the current moment time.

## 3 Experimental results

We recruited four healthy participants (four males, 23.68±0.69 years old, with heights 174.75±6.30cm and weights 76.00±10.77kg . Statistics by formula: mean ± standard deviation) to test the performance of CEARR. Appropriate approvals for this experiment were obtained from the Medical Ethics Committee of Shenzhen University Health Science Center (No. PN-202300089).

### 3.1 Experimental protocol

To assess the efficacy of CEARR system’s rehabilitation approach, the experiments are primarily categorized into two parts: testing the accuracy of the system’s BLDC motor control and verifying VTC strategy:

The ROM rehabilitation mode and performance of CEARR depend on the effective control of the BLDC motor. Only in the passive mode can isolated testing of CEARR’s BLDC motor control be conducted. Therefore, a real-time test was conducted with one participant to evaluate the control of the system’s actuators in this mode.

**FIGURE 8:**
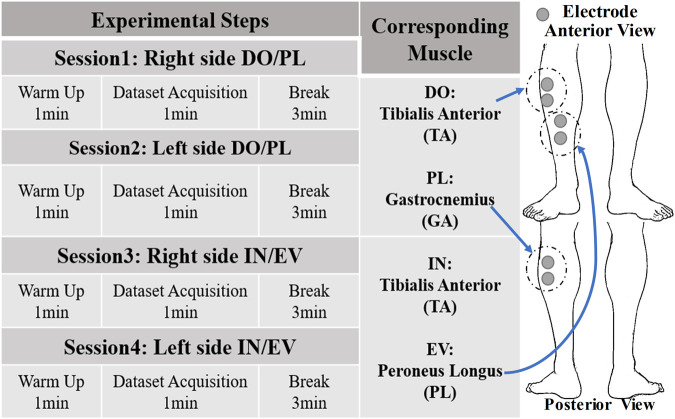
Experiment protocol of CEARR and Calf Electrode Patch Attachment Schematic.

The VTC strategy will be tested under the voluntary mode. This strategy was validated using four subjects following a unified experimental protocol. The sEMG signal acquisition locations on the muscles corresponding to the motion are depicted in Figure 8. To effectively verify the consistency between CEARR rehabilitation supports and subjects’ motion intent, an IMU (MPU9250, TDK InvenSense, USA) was employed to measure the participants’ AJC angles during motion, serving as a reference for the VTC strategy. Participants were instructed to perform four to six sets of movements over a span of 30 s while seated comfortably. Additionally, participants were asked to remain stationary for 15 s at both the beginning and end of the experiment. This approach facilitated data processing and ensured the initialization of the IMU. As previously mentioned, motion intent acquisition for AD/AB movements of the AJC cannot be accomplished using sEMG. A real-time test was also conducted using torque sensors to acquire AD/AB motion intent based on the VTC strategy, with the control performance evaluated based on these tests. To assess the VTC strategy’s performance, the coefficient of determination $R^{2}$ was employed for the analysis of motion data.

### 3.2 BLDC motors control

**FIGURE 9:**
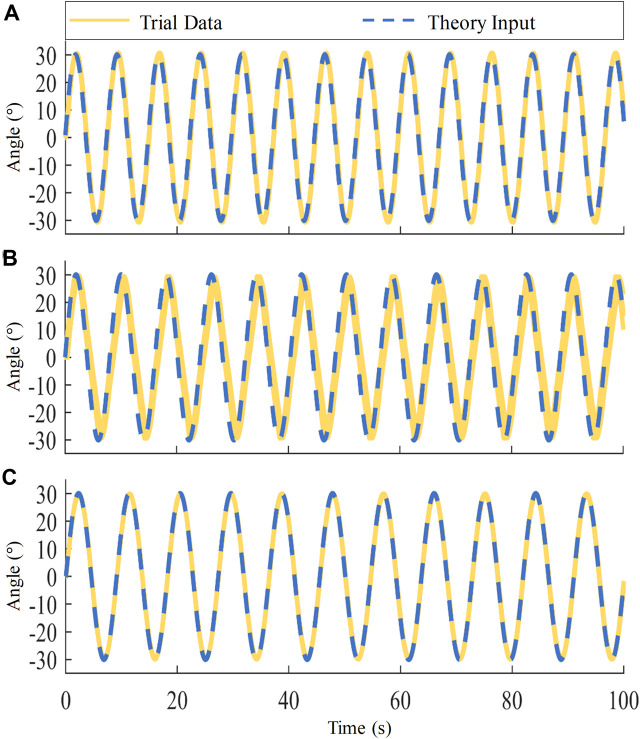
CEARR Bilateral Motor Position Error in Ankle 3-DOF during Passive Rehabilitation. **(A)** DO/PL actuator, **(B)** AD/AB actuator, **(C)** IN/EV actuator.

In this section, we input a sinusoidal signal of the similar frequency and different amplitude as the input signal for BLDC motor control based on the deceleration ratios of three different actions. Five sets of data are collected for each action, and each set of data is collected for 100 s. The theory input compared with trial angle data for each DOF actuator is shown in Figure 9. In order to assess the collaborative of bilateral motor control based on FOC in the system, the error in the motion angle of both bilateral motors during ankle motion was examined using the passive rehabilitation training mode. The results of the experiment for a single subject as shown in Table 3. The 3-DOF bilateral actuators’ mean absolute error (MAE) is 0.1257, the maximum angle error of the CEARR in the position control state is less than 1.9° , which aligns with our design expectations.

**TABLE 3:** CEARR five sets of passive mode experimental data.

Actuator	MAE ( $\left.{}^{\circ}\right.$ )	BME ( $\left.{}^{\circ}\right.$ )	Range ( $\left.{}^{\circ}\right.$ )
DO/PL	0.2089	0.2099	−30−30
AD/AB	0.7094	1.8173	
IN/EV	0.6290	0.6286	

(BME: Bilateral Maximum Error).

### 3.3 VTC strategy

In this section, the real-time performance of the VTC strategy is evaluated, based separately on sEMG and torque signals. The nonlinear sEMG signal test involves a comparison with the angular signal acquired by IMU, to verify the consistency of velocity control with the typical motion of the human AJC. Additionally, the torque signal undergoes a real-machine test.

Experimentally acquired sEMG signals were processed through a hardware bandpass filter, featuring a passband range of 23.50–448.96 Hz, from which the eigenvalues of the filtered signals were extracted. RMS feature extraction was performed on the sEMG signal using a window size of 100 sliding window. RMS feature extraction was conducted on the sEMG signals using a sliding window with a size of 100. To verify the VTC strategy’s generalizability across individuals, sEMG signals from the first subject’s DO/PL and IN/EV movements in four directions were used to determine optimal gain parameters K and a via PSO. This optimization involved 30 particles, 300 loops, inertia weights of 0.5, and both individual and group learning factors set at 1.5.

All four subjects conducted experiments using parameters derived from the optimization of the first subject’s data. The specific optimized parameters for Subject one are detailed in Table 4. To better confirm the consistency of the VTC strategy’s speed output with the actual reference output, we compare the real-time computed VTC strategy output with the IMU reference follow-up, thereby minimizing serial port communication delays and errors associated with mechanical fits. Subjects were also asked to closely follow the trajectories indicated by Unity software to complete the respective DO/PL or IN/EV motions.

**TABLE 4:** Subject1 parameters obtained from sEMG signal optimization.

Motion	Muscle	k	q
DO	TA	0.1	0.027
PL	GA	0.1	0.100
IN	TA	0.1	0.020
EV	PL	0.1	0.010

**FIGURE 10:**
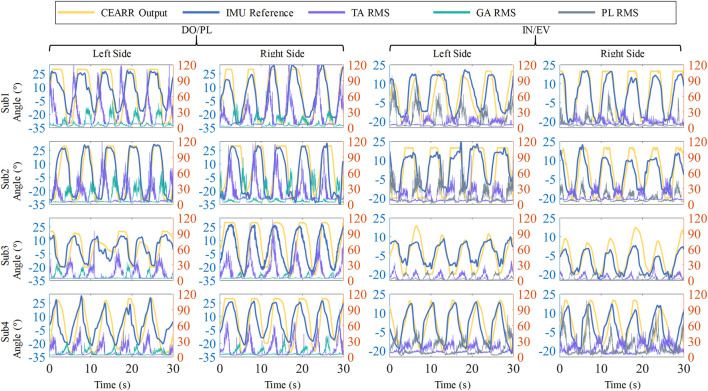
Schematic illustration of IMU reference *versus* sEMG-based VTC strategy in the CEARR, showing angular output results and sEMG RMS results for four subjects’ bilateral AJCs.

A total of 16 sets of results from the sEMG-based *versus* IMU real-time bilateral AJCs experiments for four subjects are shown in Figure 10. To further minimize the baseline error of the IMU and ensure the validity of the experimental comparison, the experiments restricted the movement angles for DO/PL to ±30° , and IN/EV to ±20° . The inclusion of the VTC strategy did not introduce a human-perceivable delay, as confirmed by the experimental results. The coefficient of determination $R^{2}$ score results for each subject’s sEMG-based analysis with IMU data are presented in Table 5.

**TABLE 5:** 16 sets of sEMG-based CEARR Motion Data with IMU $R^{2}$ Score Statistics.

Subject	DO/PL		IN/EV		Mean
	Right	Left	Right	Left	
1	0.92	0.83	0.89	0.50	0.78
2	0.73	0.91	0.67	0.70	0.75
3	0.41	0.43	0.45	0.69	0.49
4	0.41	0.65	0.70	0.79	0.64
Mean	0.62	0.70	0.68	0.67	0.67

**FIGURE 11:**
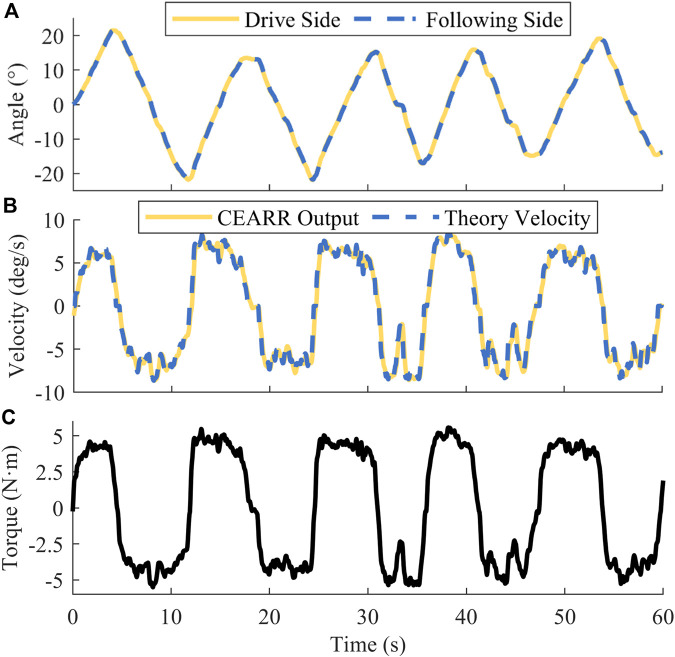
CEARR torque-based VTC strategy real-time experimental results. **(A)** Angle, **(B)** Angular velocity, **(C)** Torque.

We attempted to verify the consistency of the VTC velocity output with the linear torque signal in torque-based. In the validation of torque-based VTC control, due to the lack of mechanical analysis of Subject 3’s AJCs, the relationship between torque and angle of motion was not constructed accurately and scientifically, so we did not use PSO to train this part of the parameter, and we directly take k=50 , q=0.3 , b=ln⁡9 to let Subject three to conduct the real-time experiments. The experimental results of AD/AB bilateral collaborative mode in VTC strategy are shown in Figure 11. We amplified the voltage output of the torque sensor based on the characteristics of We amplified the voltage output of the torque sensor based on the characteristics of its internal Wheatstone bridge. We compared the torque signal with the theoretical trigger velocity and the real-time output of CEARR, and there was a high degree of agreement between the three curves, The $R^{2}$ of the torque signal with respect to the CEARR output velocity and theory velocity were 0.9821 and 0.9884, respectively.

## 4 Discussion

Foot drop and foot valgus are common limb dysfunction problems observed in stroke patients with hemiplegia, which often necessitate early ROM rehabilitation training to prevent long-term complications. The end-effector ankle rehabilitation robots, as a novel approach of rehabilitation equipment, have the potential to assist patients in surpassing the constraints posed by traditional physical therapists and engaging in a wider range of rehabilitation modalities. The limitations of current designs of end-effector robots and motion intent recognition have been outlined in Section 1.

This study developed a compact, platform-based tandem ankle rehabilitation robot system, which is designed with a compact structure and separated functions to match the needs of different patients as much as possible. The system prototype can be directly connected to the subject’s ankle without restricting other joints. The design of the tandem actuators ensures that the modification of one actuator does not affect the normal operation of other actuators. The prototype of this system adopts a minimally connected design for the connections between the actuators of each DOF, which results in a compact structural design for the CEARR system. The advantage of the tandem ankle rehabilitation robot design compared to the parallel ankle rehabilitation robot lies in the relatively easier model analysis and without singularity. CEARR ensures alignment with the ankle joint center during the concurrent operation of all four actuators. The CEARR design effectively covers all DOF pertinent to AJC, thus presenting a more holistic approach for ankle ROM rehabilitation. This design surpasses the capabilities of ankle-foot orthoses and robots limited to a single DOF (Ferris et al., 2006; Ren et al., 2017; Feng et al., 2019; Rodriguez Hernandez et al., 2023). Although the integration of an additional actuator is necessary to enable height adjustments, the incorporation of our planar motion actuator and angle adjustment bracket is designed to address this requirement effectively. The ROM rehabilitation support covers the normal range of ankle joint movement in humans and mechanical safety limit protection measures have been implemented. The prototype machine employs the FOC algorithm with torque sensor to control the BLDC motors for AD/AB motion reverse driving. The prototype machine is now functioning properly, and the motor experiments have validated its operation in the proposed rehabilitation mode. The tracking error of the bilateral motors is generally kept within 1.9° , and there is no accumulated error. This data was obtained when the motors were operating at a relatively low velocity, as stroke patients do not require a high velocity during ankle rehabilitation training. We deployed this CAD model on Unity, and based on the user’s behaviors, it exhibited good response velocity when running on the prototype machine.

To expand the rehabilitation capabilities of the CEARR, the system integrates a VTC strategy, enabling a discrete-triggered, continuous velocity control strategy driven by motion intent. This strategy is tailored to the structural characteristics of CEARR, considering the sEMG and Torque signal acquisition features. There is a lack of sufficient data on ROM rehabilitation in stroke patients with AJC issues. Moreover, machine learning or deep learning-based methods are not ideally suited for implementing an online continuous motion intent recognition strategy (Xiao et al., 2023). Unlike methods that require extensive data for training, the VTC strategy only necessitates a short initial session of forceful exercise attempts by the patient on the machine to record baseline data. This data enables the use of PSO to finetune the k and q parameters of the strategy, thus facilitating the identification of the patient’s motion intent. In order to adjust the minimum velocity support for the patient after the trigger, we can manually set the size of b to adjust the CEARR compliance. This ensures that the motor rehabilitation assistance provided is synchronized with the patient’s current AJC movement velocity. The velocity curve, formulated based on a sigmoid function, offers low velocity when RMS values are low. It avoids providing excessively high velocities for assisted movements when RMS values are high, thus offering feedback on the magnitude of the patient’s motion intent through this velocity regulation. When combined with engaging visual feedback methods, this scheme has the potential to further encourage autonomous patient participation. The VTC strategy validation experiment attempts to use the k and q parameters obtained from the optimization of the first subject to test the effect of sEMG movement in all subjects. The movement outputs of CEARR and the IMU-referenced $R^{2}$ scores, as shown in the statistics in Table 5, indicate minimal differences in motion intent consistency. Differences in AJC movements between healthy individuals and between the left and right sides were not significant. We noticed that subject 3’s data is obviously lower than the others. The data in Figure 10 clearly shows that subject 3’s ankle joint activity is not as sufficient as that of the other subjects. We believe this may be due to limitations in subject 3’s ankle joint, which could have resulted in a reduced willingness to exert force at the extreme positions of joint activity, leading to the observed decrease in experimental data.

The VTC strategy, when applied to sEMG-based healthy subjects, demonstrates generalizability. It can be readily implemented in system lower computers and later integrated with approaches like impedance control to achieve safer and more supple control outputs. However, the VTC control strategy does have its limitations, chiefly stemming from the challenges in sEMG signal acquisition. Reliance on single muscle sEMG values for distinct movements might lead to incorrect recognition of movement intent, especially during simultaneous movements in multiple directions, like DO and EV. sEMG faces challenges in effectively capturing useful signals. Additionally, a suitable force-position relationship must be established prior to employing force signals in the VTC strategy. Furthermore, the torque signal, being a direct force signal, does not present the issue of incorrect movement intention recognition, a design consideration in the CEARR. Utilizing BLDC motors and VTC strategy, the CEARR design demonstrates a markedly rapid response time, significantly outperforming pneumatic drive methods (Girone et al., 2001; Wang et al., 2022), with an observed average the CEARR system response time falling below 300 milliseconds.

In future research, clinical trials will be conducted to validate the rehabilitation efficacy of this system in stroke patients. Additionally, it has been observed that the wired sEMG acquisition setup on the lower limbs can be cumbersome. Hence, there are plans to optimize the signal acquisition method by transitioning to wireless acquisition, effectively eliminating artifacts caused by cable interference. Efforts will also be made to employ a machine learning-based continuous identification method for performance comparison with the VTC strategy, specifically in terms of velocity change control algorithms driven by motion intent.

## 5 Conclusion

This article proposed and developed a novel, compact tandem ankle rehabilitation robotic system for three degrees of freedom range of motion ankle rehabilitation in both seated and bedridden postures. The prototype of the system compactly integrates a planar motion actuator with a foldable adjustment bracket on its platform. This design ensured versatile positional adjustments, supporting patients in both seated and supine positions, while its compact structure facilitated rehabilitation in a variety of scenarios. The actuators are independent and designed to be connected in series, allowing for easy structural adjustments and improvements. The use of BLDC motor drive and a rack-and-pinion engagement approach enables the CEARR to achieve faster response and precise angle control. By analyzing sEMG and torque signals during the rehabilitation process, the system offered three specialized rehabilitation modes, including passive, bilateral collaboration, and voluntary, and each mode was tailored to cater to stroke patients in varying situations, based on ROM rehabilitation. A voluntary-based motion intent strategy has been developed to enhance the execution of these rehabilitation modalities. This strategy is cost-effective as it can be implemented on a single microcontroller, unlike neural network-based algorithms for movement intention prediction, which are highly demanding in terms of computational resources. The system and this strategy underwent real-time control testing with four subjects, and their performance met the design expectations. CEARR performs well in both response time and ROM rehabilitation support.

## Data Availability

The original contributions presented in the study are included in the article/supplementary material, further inquiries can be directed to the corresponding authors.
